# Beyond phage display: non-traditional applications of the filamentous bacteriophage as a vaccine carrier, therapeutic biologic, and bioconjugation scaffold

**DOI:** 10.3389/fmicb.2015.00755

**Published:** 2015-08-04

**Authors:** Kevin A. Henry, Mehdi Arbabi-Ghahroudi, Jamie K. Scott

**Affiliations:** ^1^Human Health Therapeutics Portfolio, National Research Council Canada, OttawaON, Canada; ^2^School of Environmental Sciences, University of Guelph, GuelphON, Canada; ^3^Department of Biology, Carleton University, OttawaON, Canada; ^4^Department of Molecular Biology and Biochemistry, Simon Fraser University, Burnaby, BCCanada; ^5^Faculty of Health Sciences, Simon Fraser University, BurnabyBC, Canada

**Keywords:** filamentous phage, vaccine, therapeutic, antimicrobial, bioconjugation

## Abstract

For the past 25 years, phage display technology has been an invaluable tool for studies of protein–protein interactions. However, the inherent biological, biochemical, and biophysical properties of filamentous bacteriophage, as well as the ease of its genetic manipulation, also make it an attractive platform outside the traditional phage display canon. This review will focus on the unique properties of the filamentous bacteriophage and highlight its diverse applications in current research. Particular emphases are placed on: (i) the advantages of the phage as a vaccine carrier, including its high immunogenicity, relative antigenic simplicity and ability to activate a range of immune responses, (ii) the phage’s potential as a prophylactic and therapeutic agent for infectious and chronic diseases, (iii) the regularity of the virion major coat protein lattice, which enables a variety of bioconjugation and surface chemistry applications, particularly in nanomaterials, and (iv) the phage’s large population sizes and fast generation times, which make it an excellent model system for directed protein evolution. Despite their ubiquity in the biosphere, metagenomics work is just beginning to explore the ecology of filamentous and non-filamentous phage, and their role in the evolution of bacterial populations. Thus, the filamentous phage represents a robust, inexpensive, and versatile microorganism whose bioengineering applications continue to expand in new directions, although its limitations in some spheres impose obstacles to its widespread adoption and use.

## Introduction

The filamentous bacteriophage (genera *Inovirus* and *Plectrovirus*) are non-enveloped, rod-shaped viruses of *Escherichia coli* whose long helical capsids encapsulate a single-stranded circular DNA genome. Subsequent to the independent discovery of bacteriophage by Twort (1915) and d’Hérelle (1917), the first filamentous phage, f1, was isolated in Loeb (1960) and later characterized as a member of a larger group of phage (Ff, including f1, M13, and fd phage) specific for the *E. coli* conjugative F pilus (Hofschneider and Mueller-Jensen, 1963; Marvin and Hoffmann-Berling, 1963; Zinder et al., 1963; Salivar et al., 1964). Soon thereafter, filamentous phage were discovered that do not use F-pili for entry (If and Ike; Meynell and Lawn, 1968; Khatoon et al., 1972), and over time the list of known filamentous phage has expanded to over 60 members (Fauquet et al., 2005), including temperate and Gram-positive-tropic species. Work by multiple groups over the past 50 years has contributed to a relatively sophisticated understanding of filamentous phage structure, biology and life cycle (reviewed in Marvin, 1998; Rakonjac et al., 2011; Rakonjac, 2012).

In the mid-1980s, the principle of modifying the filamentous phage genome to display polypeptides as fusions to coat proteins on the virion surface was invented by Smith and colleagues (Smith, 1985; Parmley and Smith, 1988). Based on the ideas described in Parmley and Smith (1988), groups in California, Germany, and the UK developed phage-display platforms to create and screen libraries of peptide and folded-protein variants (Bass et al., 1990; Devlin et al., 1990; McCafferty et al., 1990; Scott and Smith, 1990; Breitling et al., 1991; Kang et al., 1991). This technology allowed, for the first time, the ability to seamlessly connect genetic information with protein function for a large number of protein variants simultaneously, and has been widely and productively exploited in studies of protein–protein interactions. Many excellent reviews are available on phage-display libraries and their applications (Kehoe and Kay, 2005; Bratkovic, 2010; Pande et al., 2010). However, the phage also has a number of unique structural and biological properties that make it highly useful in areas of research that have received far less attention.

Thus, the purpose of this review is to highlight recent and current work using filamentous phage in novel and non-traditional applications. Specifically, we refer to projects that rely on the filamentous phage as a key element, but whose primary purpose is not the generation or screening of phage-displayed libraries to obtain binding polypeptide ligands. These tend to fall into four major categories of use: (i) filamentous phage as a vaccine carrier; (ii) engineered filamentous phage as a therapeutic biologic agent in infectious and chronic diseases; (iii) filamentous phage as a scaffold for bioconjugation and surface chemistry; and (iv) filamentous phage as an engine for evolving variants of displayed proteins with novel functions. A final section is dedicated to recent developments in filamentous phage ecology and phage–host interactions. Common themes shared amongst all these applications include the unique biological, immunological, and physicochemical properties of the phage, its ability to display a variety of biomolecules in modular fashion, and its relative simplicity and ease of manipulation.

## Filamentous Phage Display Systems: An Overview

Nearly all applications of the filamentous phage depend on its ability to display polypeptides on the virion’s surface as fusions to phage coat proteins (**Table 1**). The display mode determines the maximum tolerated size of the fused polypeptide, its copy number on the phage, and potentially, the structure of the displayed polypeptide. Display may be achieved by fusing DNA encoding a polypeptide of interest directly to the gene encoding a coat protein within the phage genome (type 8 display on pVIII, type 3 display on pIII, etc.), resulting in fully recombinant phage. Much more commonly, however, only one copy of the coat protein is modified in the presence of a second, wild-type copy (e.g., type 88 display if both recombinant and wild-type pVIII genes are on the phage genome, type 8+8 display if the recombinant gene 8 is on a plasmid with a phage origin of replication) resulting in a hybrid virion bearing two different types of a given coat protein. Multivalent display on some coat proteins can also be enforced using helper phage bearing non-functional copies of the relevant coat protein gene (e.g., type 3^∗^+3 display). By far the most commonly used coat proteins for display are the major coat protein, pVIII, and the minor coat protein, pIII, with the major advantage of the former being higher copy number display (up to ~15% of recombinant pVIII molecules in a hybrid virion, at least for short peptide fusions), and of the latter being the ability to display some folded proteins at an appreciable copy number (1–5 per phage particle). While pVIII display of folded proteins on hybrid phage is possible, it typically results in a copy number of much less than 1 per virion (Sidhu et al., 2000). For the purposes of this review, we use the term “phage display” to refer to a recombinant filamentous phage displaying a single polypeptide sequence on its surface (or more rarely, bispecific display achieved *via* fusion of polypeptides to two different capsid proteins), and the term “phage-displayed library” to refer to a diverse pool of recombinant filamentous phage displaying an array of polypeptide variants (e.g., antibody fragments; peptides). Such libraries are typically screened by iterative cycles of panning against an immobilized protein of interest (e.g., antigen for phage-displayed antibody libraries; antibody for phage-displayed peptide libraries) followed by amplification of the bound phage in *E. coli* cells.

**Table 1:** Filamentous phage display modes and their associated characteristics.

Phage coat protein	Display mode	Helper phage required?	Polypeptide copy number	Polypeptide size limit	Reference(s)
pIII	Fully recombinant (type 3 and 3+3^∗^ systems)^1^	Type 3+3^∗^ system	~5	>25 kDa	Parmley and Smith (1988), McConnell et al. (1994),Rondot et al. (2001)
	Hybrid (type 33 and 3+3 systems)	Type 3+3 system	<1^2^		Smith and Scott (1993),Smith and Petrenko (1997)
pVI	Hybrid (type 6+6 system)	Yes	<1^2^	>25 kDa	Hufton et al. (1999)
pVII	Fully recombinant (type 7 system)	No	~5	>25 kDa	Kwasnikowski et al. (2005)
	Hybrid (type 7+7 system)	Yes	<1^2^		Gao et al. (1999)
pVIII	Fully recombinant (landscape phage; type 8 system)	No	2700^3^	~5–8 residues	Kishchenko et al. (1994), Petrenko et al. (1996)
	Hybrid (type 88 and 8+8 systems)	Type 8+8 system	~1–300^2^	>50 kDa	Scott and Smith (1990), Greenwood et al. (1991),Smith and Fernandez (2004)
pIX	Fully recombinant (type 9+9^∗^ system)	Yes	~5	>25 kDa	Gao et al. (2002)
	Hybrid (type 9+9 system)	No	<1^2^		Gao et al. (1999), Shi et al. (2010), Tornetta et al. (2010)

*^1^Asterisks indicate non-functional copies of the coat protein are present in the genome of the helper phage used to rescue a phagemid whose coat protein has been fused to a recombinant polypeptide.*

*^2^The copy number depends on polypeptide size; typically <1 copy per phage particle but for pVIII peptide display can be up to ~15% of pVIII molecules in hybrid virions.*

*^3^The total number of pVIII molecules depends on the phage genome size; one pVIII molecule is added for every 2.3 nucleotides in the viral genome.*

## Filamentous Phage as an Immunogenic Vaccine Carrier

Early work with anti-phage antisera generated for species classification purposes demonstrated that the filamentous phage virion is highly immunogenic in the absence of adjuvants (Meynell and Lawn, 1968) and that only the major coat protein, pVIII, and the minor coat protein, pIII, are targeted by antibodies (Pratt et al., 1969; Woolford et al., 1977). Thus, the idea of using the phage as carrier to elicit antibodies against poorly immunogenic haptens or polypeptide was a natural extension of the ability to display recombinant exogenous sequences on its surface, which was first demonstrated by de la Cruz et al. (1988). The phage particle’s low cost of production, high stability and potential for high valency display of foreign antigen (*via* pVIII display) also made it attractive as a vaccine carrier, especially during the early stages of development of recombinant protein technology.

### Antibody Epitope-Based Peptide Vaccines

Building upon existing peptide-carrier technology, the first filamentous phage-based vaccine immunogens displayed short amino acid sequences derived directly from proteins of interest as recombinant fusions to pVIII or pIII (de la Cruz et al., 1988). As library technology was developed and refined, phage-based antigens displaying peptide ligands of monoclonal antibodies (selected from random peptide libraries using the antibody, thus simulating with varying degrees of success the antibody’s folded epitope on its cognate antigen; Geysen et al., 1986; Knittelfelder et al., 2009) were also generated for immunization purposes, with the goal of eliciting anti-peptide antibodies that also recognize the native protein. Some of the pioneering work in this area used peptides derived from infectious disease antigens (or peptide ligands of antibodies against these antigens; **Table 2**), including malaria and human immunodeficiency virus type 1 (HIV-1). When displayed on phage, peptides encoding the repeat regions of the malarial circumsporozoite protein and merozoite surface protein 1 were immunogenic in mice and rabbits (de la Cruz et al., 1988; Greenwood et al., 1991; Willis et al., 1993; Demangel et al., 1996), and antibodies raised against the latter cross-reacted with the full-length protein. Various peptide determinants (or mimics thereof) of HIV-1 gp120, gp41, gag, and reverse transcriptase were immunogenic when displayed on or conjugated to phage coat proteins (Minenkova et al., 1993; di Marzo Veronese et al., 1994; De Berardinis et al., 1999; Scala et al., 1999; Chen et al., 2001; van Houten et al., 2006, 2010), and in some cases elicited antibodies that were able to weakly neutralize lab-adapted viruses (di Marzo Veronese et al., 1994; Scala et al., 1999). The list of animal and human infections for which phage-displayed peptide immunogens have been developed as vaccine leads continues to expand and includes bacterial, fungal, viral, and parasitic pathogens (**Table 2**). While in some cases the results of these studies have been promising, antibody epitope-based peptide vaccines are no longer an area of active research for several reasons: (i) in many cases, peptides incompletely or inadequately mimic epitopes on folded proteins (Irving et al., 2010; see below); (ii) antibodies against a single epitope may be of limited utility, especially for highly variable pathogens (Van Regenmortel, 2012); and (iii) for pathogens for which protective immune responses are generated efficiently during natural infection, peptide vaccines offer few advantages over recombinant subunit and live vector vaccines, which have become easier to produce over time.

**Table 2:** Studies using filamentous phage as an immunogenic carrier for peptide B-cell epitopes.

Antigen	Species	Epitope type(s)	Display format(s)	Ab response against peptide	Ab response against native protein	Reference(s)
Alzheimer’s disease β-amyloid fibrils	Mouse (BALB/c, APP transgenic), Guinea pig	Linear	pVIII hybrid, pIII fully recombinant	+	+	Frenkel et al. (2000, 2003), Lavie et al. (2004), Esposito et al. (2008), Tanaka et al. (2011)
*Aspergillus fumigatus* MIRB	Mouse (BALB/c)	Linear	Chemical conjugate	+	+	Raymond-Bouchard et al. (2012)
*Bordetella pertussis* toxin	Mouse (BALB/c)	Discontinuous	pVIII hybrid	+	-	Felici et al. (1993)
*Candida albicans* HSP90	Mouse (C57/BL6, BALB/c)	Linear	pVIII hybrid	+	+	Yang et al. (2005a), Wang et al. (2006, 2014d)
*Chlamydia trachomatis* major outer membrane protein	Mouse (C57/BL6, BALB/c, CBA/j)	Linear	pVIII hybrid	+	+	Zhong et al. (1994)
*Coronavirus S* glycoprotein	Mouse (C57/BL6, BALB/c)	Linear	pVIII hybrid	+	+	Yu et al. (2000)
ERBB2/HER2	Mouse (BALB/c)	Linear	pVIII hybrid, pIII fully recombinant	+	+	Yip et al. (2001)
*Entamoeba histolytica* proteophosphoglycan	Mouse (BALB/c)	Discontinuous	pVIII hybrid	+	+	Melzer et al. (2002)
Follicle-stimulating hormone	Sheep	Linear	pVIII hybrid	+	+	Abdennebi et al. (1999)
Foot-and-mouth disease VP1	Guinea pig	Linear	pVIII hybrid	ND	+	Kim et al. (2004)
Gonadotropin-releasing hormone	Mouse (CD-1)	Linear	Chemical conjugate	ND	ND	Samoylov et al. (2012)
HBV surface antigen	Mouse (C57/BL6, BALB/c, B10.M)	Linear	pVIII hybrid, pIII fully recombinant	+	+	Folgori et al. (1994), Motti et al. (1994), Meola et al. (1995), Delmastro et al. (1997)
HCV core antigen and NS4B protein	Mouse (C57/BL6, BALB/c, MF1)	Linear	pVIII hybrid	+	ND	Prezzi et al. (1996), Delmastro et al. (1997)
HCV E2 protein	Mouse (BALB/c)	Linear	pVIII hybrid	+	ND	Puntoriero et al. (1998)
*Helicobacter pylori*Urease B	Mouse (BALB/c)	Linear	pIII fully recombinant	+	+	Li et al. (2010c)
HIV-1 gp120 V3 loop	Mouse (BALB/c)	Linear	pVIII hybrid	+	+	di Marzo Veronese et al. (1994)
HIV-1 gp120 CD4-binding site	Mouse (BALB/c)	Discontinuous	Chemical conjugate	+	-	van Houten et al. (2006)
HIV-1 gp120 and gp41	Mouse (C57/BL6, BALB/c), *Rhesus macaque*	Linear, Discontinuous	pVIII hybrid, pIII hybrid, Chemical conjugate	+	ND	Scala et al. (1999), Chen et al. (2001), van Houten et al. (2010)
HIV-1 gag p17	Rabbit	Linear	pVIII fully recombinant	+	+	Minenkova et al. (1993)
HIV-1 reverse transcriptase	Mouse (C57BL/6, HLA-A2 transgenic)	Linear	pVIII hybrid	+	ND	De Berardinis et al. (1999)
HPV E7 protein	Mouse (BALB/c)	Linear	pVIII hybrid	+	+	Lidqvist et al. (2008)
HSV-2 glycoprotein G	Mouse (BALB/c)	Linear	pVIII hybrid	+	+	Grabowska et al. (2000)
Immunoglobulin E	Rabbit	Discontinuous	pVIII hybrid	ND	+	Rudolf et al. (1998)
*Mycoplasma hyopneumoniae* unknown proteins	Mouse (BALB/cByJ)	Linear, Discontinuous	pIII fully recombinant	+	+	Yang et al. (2005b)
*Neisseria meningitidis* PorA protein	Mouse (BALB/c)	Linear	pVIII hybrid	+	+	Menendez et al. (2001)
*Plasmodium falciparum* CSP	Mouse (BALB/c, C57/BL10, Swiss, Str/ort), rabbit	Linear	pVIII hybrid, pIII fully recombinant	+	ND	de la Cruz et al. (1988), Greenwood et al. (1991), Willis et al. (1993)
*Plasmodium vivax* MSP1	Mouse (C57/BL6, BALB/c, Biozzi)	Linear, Discontinuous	pIII hybrid	ND	+	Demangel et al. (1996)
Rhabdovirus RABVG	Mouse (BALB/c)	Linear	pIII fully recombinant	+	+	Houimel and Dellagi (2009)
RSV glycoprotein G	Mouse (BALB/c)	Linear	pIII fully recombinant	+	-	Bastien et al. (1997)
*Schistosoma japonicum* unknown protein(s)	Mouse (Kunming)	Discontinuous	pIII fully recombinant	+	ND	
*Shigella flexneri* LPS	Mouse (BALB/c)	Discontinuous	pVIII hybrid, Chemical conjugate	+	-	van Houten et al. (2010), Henry et al. (2011)
Sperm	Mouse (CD-1), Pig	Discontinuous	pVIII fully recombinant	+	+	Samoylova et al. (2012a,b)
Toxic shock syndrome toxin	Mouse (BALB/c)	Linear	pIII fully recombinant	+	ND	Rubinchik and Chow (2000)

More recently, peptide-displaying phage have been used in attempts to generate therapeutic antibody responses for chronic diseases, cancer, immunotherapy, and immunocontraception. Immunization with phage displaying Alzheimer’s disease β-amyloid fibril peptides elicited anti-aggregating antibodies in mice and guinea pigs (Frenkel et al., 2000, 2003; Esposito et al., 2008; Tanaka et al., 2011), possibly reduced amyloid plaque formation in mice (Frenkel et al., 2003; Solomon, 2005; Esposito et al., 2008), and may have helped maintain cognitive abilities in a transgenic mouse model of Alzheimer’s disease (Lavie et al., 2004); however, it remains unclear how such antibodies are proposed to cross the blood–brain barrier. Yip et al. (2001) found that antibodies raised in mice against an ERBB2/HER2 peptide could inhibit breast-cancer cell proliferation. Phage displaying peptide ligands of an anti-IgE antibody elicited antibodies that bound purified IgE molecules (Rudolf et al., 1998), which may be useful in allergy immunotherapy. Several strategies for phage-based contraceptive vaccines have been proposed for control of animal populations. For example, immunization with phage displaying follicle-stimulating hormone peptides on pVIII elicited antibodies that impaired the fertility of mice and ewes (Abdennebi et al., 1999). Phage displaying or chemically conjugated to sperm antigen peptides or peptide mimics (Samoylova et al., 2012a,b) and gonadotropin-releasing hormone (Samoylov et al., 2012) are also in development.

For the most part, peptides displayed on phage elicit antibodies in experimental animals (**Table 2**), although this depends on characteristics of the peptide and the method of its display: pIII fusions tend toward lower immunogenicity than pVIII fusions (Greenwood et al., 1991) possibly due to copy number differences (pIII: 1–5 copies vs. pVIII: estimated at several hundred copies; Malik et al., 1996). In fact, the phage is at least as immunogenic as traditional carrier proteins such as bovine serum albumin (BSA) and keyhole limpet hemocyanin (KLH; Melzer et al., 2003; Su et al., 2007), and has comparatively few endogenous B-cell epitopes to divert the antibody response from its intended target (Henry et al., 2011). Excepting small epitopes that can be accurately represented by a contiguous short amino acid sequence, however, it has been extremely difficult to elicit antibody responses that cross-react with native protein epitopes using peptides. The overall picture is considerably bleaker than that painted by **Table 2**, since in several studies either: (i) peptide ligands selected from phage-displayed libraries were classified by the authors as mimics of discontinuous epitopes if they bore no obvious sequence homology to the native protein, which is weak evidence of non-linearity, or (ii) the evidence for cross-reactivity of antibodies elicited by immunization with phage-displayed peptides with native protein was uncompelling. Irving et al. (2010) describe at least one reason for this lack of success: it seems that peptide antigens elicit a set of topologically restricted antibodies that are largely unable to recognize discontinuous or complex epitopes on larger biomolecules. While the peptide may mimic the chemistry of a given epitope on a folded protein (allowing it to cross-react with a targeted antibody), being a smaller molecule, it cannot mimic the topology of that antibody’s full epitope. Despite this, the filamentous phage remains highly useful as a carrier for peptides with relatively simple secondary structures, which may be stablilized *via* anchoring to the coat proteins (Henry et al., 2011). This may be especially true of peptides with poor inherent immunogenicity, which may be increased by high-valency display and phage-associated adjuvanticity (see Immunological Mechanisms of Vaccination with Filamentous Phage below).

### Cytotoxic T-Cell-Based Vaccines

The filamentous phage has been used to a lesser extent as a carrier for T-cell peptide epitopes, primarily as fusion proteins with pVIII (**Table 3**). Early work, showing that immunization with phage elicited T-cell help (Kölsch et al., 1971; Willis et al., 1993), was confirmed by several subsequent studies (De Berardinis et al., 1999; Ulivieri et al., 2008). From the perspective of vaccination against infectious disease, De Berardinis et al. (2000) showed that a cytotoxic T-cell (CTL) epitope from HIV-1 reverse transcriptase could elicit antigen-specific CTLs *in vitro* and *in vivo* without addition of exogenous helper T-cell epitopes, presumably since these are already present in the phage coat proteins (Mascolo et al., 2007). Similarly, efficient priming of CTLs was observed against phage-displayed T-cell epitopes from Hepatitis B virus (Wan et al., 2001) and *Candida albicans* (Yang et al., 2005a; Wang et al., 2006, 2014d), which, together with other types of immune responses, protected mice against systemic candidiasis. Vaccination with a combination of phage-displayed peptides elicited antigen-specific CTLs that proved effective in reducing porcine cysticercosis in a randomized controlled trial (Manoutcharian et al., 2004; Morales et al., 2008).

**Table 3:** Studies using filamentous phage as an immunogenic carrier for peptide T-cell epitopes.

Antigen	Species	Epitope type(s)	Display format(s)	T-cell response against peptide	Reference(s)
*Taenia crassiceps* KETc1, KETc7, and KETc12	Pig	MHC class I	pVIII hybrid, pIII fully recombinant	+	Manoutcharian et al. (2004), Morales et al. (2008)
Ovalbumin	Mouse (C57BL/6)	MHC class I	pVIII hybrid	+	Mascolo et al. (2007)
HIV-1 reverse transcriptase	Mouse (C57BL/6, HLA-A2 transgenic)	MHC class I, MHC class II	pVIII hybrid	+	De Berardinis et al. (1999, 2000)
PIA tumor antigen	Mouse (DBA/2)	MHC class I	pVIII hybrid	+	Wu et al. (2002)
Ovalbumin (B16.OVA model)	Mouse (C57BL/6, tlr4^-/-^, OT-I)	MHC class I	pVIII hybrid	+	Sartorius et al. (2011)
MAGE-A1, MAGE-A10, MAGE-A3	Mouse (C57BL/6, HLA-A2 transgenic)	MHC class I	pVIII hybrid	+	Fang et al. (2005), Sartorius et al. (2008)
*Candida albicans* HSP90	Mouse (C57BL6/, BALB/c)	MHC class I, MHC class II	pVIII hybrid	+	Yang et al. (2005a), Wang et al. (2006, 2014d)
HBV	Mouse (BALB/c)	MHC class I	pVIII hybrid	+	Wan et al. (2001)

While the correlates of vaccine-induced immune protection for infectious diseases, where they are known, are almost exclusively serum or mucosal antibodies (Plotkin, 2010), a primary goal of cancer vaccines is to elicit therapeutic CTL responses and/or suppress CD4^+^ regulatory T cells against tumor antigens while avoiding triggering adverse autoimmune responses. To this end, Wu et al. (2002) showed that a phage-displayed CTL epitope derived from the P1A tumor antigen was immunogenic and that the CTLs elicited in response to immunization both prevented mastocytoma tumor establishment and prolonged survival in mice with established tumors. Similarly, melanoma tumor CTL epitopes (MAGE-A1, MAGE-A3, and MAGE-A10) elicited CTLs that slowed tumor growth in mice and prolonged their survival (Fang et al., 2005; Sartorius et al., 2008). This work was later extended by Sartorius et al. (2011), who showed using the B16-OVA tumor model that phage double-displaying an ovalbumin CTL epitope on pVIII and an scFv against DEC-205 (a dendritic cell marker) on pIII could elicit much stronger anti-B16 tumor CTL responses that further improved survival rates. Thus, despite their large size, filamentous phage particles can elicit vigorous CTL responses, which can be enhanced in some cases by direct targeting of the phage to antigen-presenting cells. Although no studies have conducted a thorough comparison of the immunogenicity of a single CTL epitope in the context of different carriers, it is possible that the filamentous phage virion may induce stronger and more durable CTL responses compared to soluble exogenous proteins because of its efficient cross-presentation into the MHC class I pathway (see Immunological Mechanisms of Vaccination with Filamentous Phage below).

### Recombinant Protein and DNA Vaccines

In certain vaccine applications, the filamentous phage has been used as a carrier for larger molecules that would be immunogenic even in isolation. Initially, the major advantages to phage display of such antigens were speed, ease of purification and low cost of production (Gram et al., 1993). *E. coli* F17a-G adhesin (Van Gerven et al., 2008), hepatitis B core antigen (Bahadir et al., 2011), and hepatitis B surface antigen (Balcioglu et al., 2014) all elicited antibody responses when displayed on pIII, although none of these studies compared the immunogenicity of the phage-displayed proteins with that of the purified protein alone. Phage displaying *Schistosoma mansoni* glutathione *S*-transferase on pIII elicited an antibody response that was both higher in titer and of different isotypes compared to immunization with the protein alone (Rao et al., 2003). Two studies of anti-idiotypic vaccines have used the phage as a carrier for antibody fragments bearing immunogenic idiotypes. Immunization with phage displaying the 1E10 idiotype scFv (mimicking a *Vibrio anguillarum* surface epitope) elicited antibodies that protected flounder fish from *Vibrio anguillarum* challenge (Xia et al., 2005). A chemically linked phage-BCL1 tumor-specific idiotype vaccine was weakly immunogenic in mice but extended survival time in a B-cell lymphoma model (Roehnisch et al., 2013), and was well-tolerated and immunogenic in patients with multiple myeloma (Roehnisch et al., 2014). One study of DNA vaccination with an anti-laminarin scFv found that DNA encoding a pIII-scFv fusion protein elicited stronger humoral and cell-mediated immune responses than DNA encoding the scFv alone (Cuesta et al., 2006), suggesting that under some circumstances, endogenous phage T-cell epitopes can enhance the immunogenicity of associated proteins. Taken together, the results of these studies show that as a particulate virus-like particle, the filamentous phage likely triggers different types of immune responses than recombinant protein antigens, and provide additional T-cell help to displayed or conjugated proteins. However, the low copy number of pIII-displayed proteins, as well as potentially unwanted phage-associated adjuvanticity, can make display of recombinant proteins by phage a suboptimal vaccine choice.

### Immunological Mechanisms of Vaccination with Filamentous Phage

**FIGURE 1:**
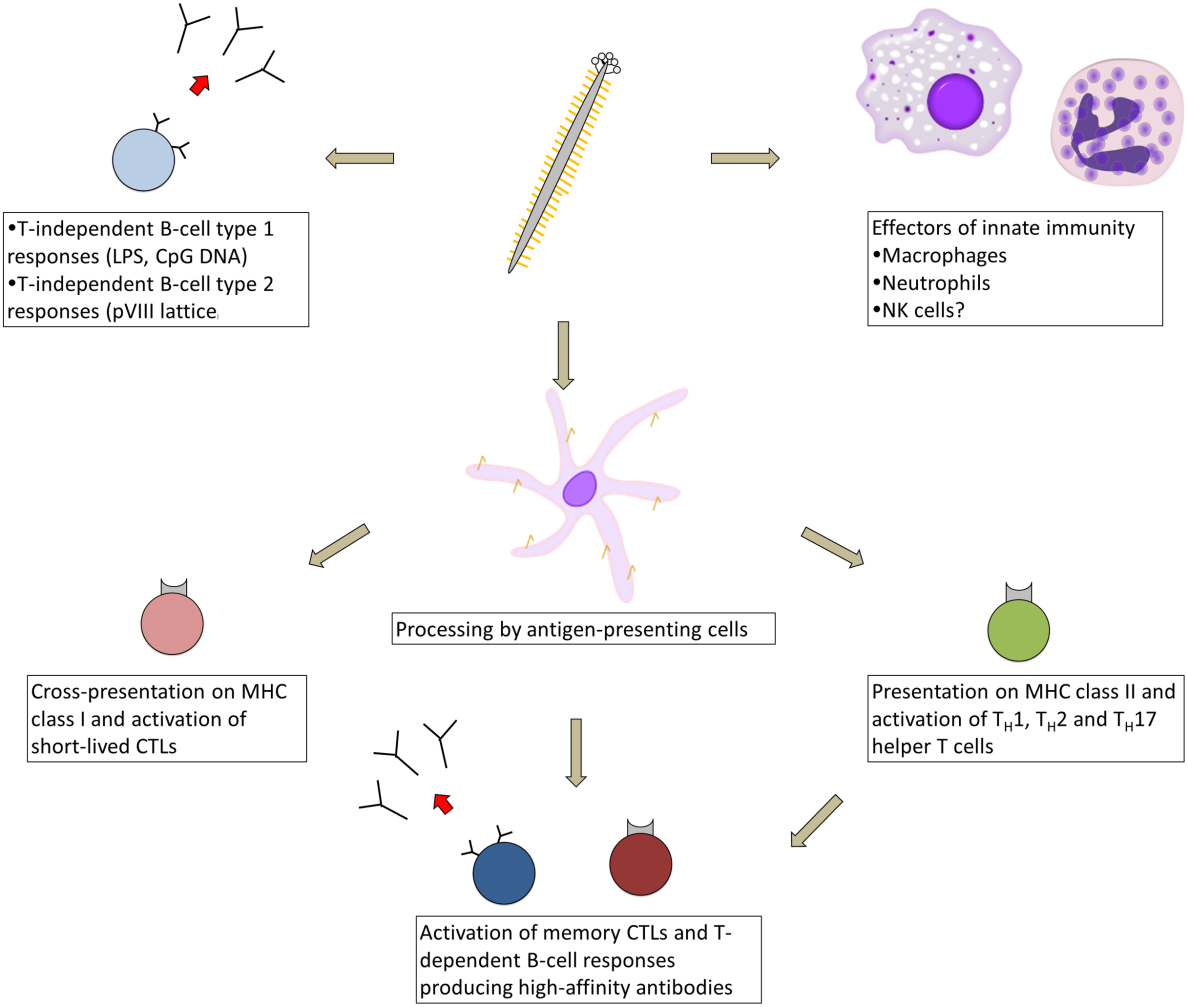
**Types of immune responses elicited in response to immunization with filamentous bacteriophage.** As a virus-like particle, the filamentous phage engages multiple arms of the immune system, beginning with cellular effectors of innate immunity (macrophages, neutrophils, and possibly natural killer cells), which are recruited to tumor sites by phage displaying tumor-targeting moieties. The phage likely activates T-cell independent antibody responses, either via phage-associated TLR ligands or cross-linking by the pVIII lattice. After processing by antigen-presenting cells, phage-derived peptides are presented on MHC class II and cross-presented on MHC class I, resulting in activation of short-lived CTLs and an array of helper T-cell types, which help prime memory CTL and high-affinity B-cell responses.

Although our understanding of the immune response against the filamentous phage pales in comparison to classical model antigens such as ovalbumin, recent work has begun to shed light on the immune mechanisms activated in response to phage vaccination (**Figure 1**). The phage particle is immunogenic without adjuvant in all species tested to date, including mice (Willis et al., 1993), rats (Dente et al., 1994), rabbits (de la Cruz et al., 1988), guinea pigs (Frenkel et al., 2000; Kim et al., 2004), fish (Coull et al., 1996; Xia et al., 2005), non-human primates (Chen et al., 2001), and humans (Roehnisch et al., 2014). Various routes of immunization have been employed, including oral administration (Delmastro et al., 1997) as well as subcutaneous (Grabowska et al., 2000), intraperitoneal (van Houten et al., 2006), intramuscular (Samoylova et al., 2012a), intravenous (Vaks and Benhar, 2011), and intradermal injection (Roehnisch et al., 2013); no published study has directly compared the effect of administration route on filamentous phage immunogenicity. Antibodies are generated against only three major sites on the virion: (i) the surface-exposed N-terminal ~12 residues of the pVIII monomer lattice (Terry et al., 1997; Kneissel et al., 1999); (ii) the N-terminal N1 and N2 domains of pIII (van Houten et al., 2010); and (iii) bacterial lipopolysaccharide (LPS) embedded in the phage coat (Henry et al., 2011). In mice, serum antibody titers against the phage typically reach 1:10^5^–1:10^6^ after 2–3 immunizations, and are maintained for at least 1 year post-immunization (Frenkel et al., 2000). Primary antibody responses against the phage appear to be composed of a mixture of IgM and IgG2b isotypes in C57BL/6 mice, while secondary antibody responses are composed primarily of IgG1 and IgG2b isotypes, with a lesser contribution of IgG2c and IgG3 isotypes (Hashiguchi et al., 2010). Deletion of the surface-exposed N1 and N2 domains of pIII produces a truncated form of this protein that does not elicit antibodies, but also results in a non-infective phage particle with lower overall immunogenicity (van Houten et al., 2010).

Although serum anti-phage antibody titers appear to be at least partially T-cell dependent (Kölsch et al., 1971; Willis et al., 1993; De Berardinis et al., 1999; van Houten et al., 2010), many circulating pVIII-specific B cells in the blood are devoid of somatic mutation even after repeated biweekly immunizations, suggesting that under these conditions, the phage activates T-cell-independent B-cell responses in addition to high-affinity T-cell-dependent responses (Murira, 2014). Filamentous phage particles can be processed by antigen-presenting cells and presented on MHC class II molecules (Gaubin et al., 2003; Ulivieri et al., 2008) and can activate T_H_1, T_H_2, and T_H_17 helper T cells (Yang et al., 2005a; Wang et al., 2014d). Anti-phage T_H_2 responses were enhanced through display of CTLA-4 peptides fused to pIII (Kajihara et al., 2000). Phage proteins can also be cross-presented on MHC class I molecules (Wan et al., 2005) and can prime two waves of CTL responses, consisting first of short-lived CTLs and later of long-lived memory CTLs that require CD4^+^ T-cell help (Del Pozzo et al., 2010). The latter CTLs mediate a delayed-type hypersensitivity reaction (Fang et al., 2005; Del Pozzo et al., 2010).

The phage particle is self-adjuvanting through multiple mechanisms. Host cell wall-derived LPS enhances the virion’s immunogenicity, and its removal by polymyxin B chromatography reduces antibody titers against phage coat proteins (Grabowska et al., 2000). The phage’s single-stranded DNA genome contains CpG motifs and may also have an adjuvant effect. The antibody response against the phage is entirely dependent on MyD88 signaling and is modulated by stimulation of several Toll-like receptors (Hashiguchi et al., 2010), indicating that innate immunity plays an important but largely uncharacterized role in the activation of anti-phage adaptive immune responses. Biodistribution studies of the phage after intravenous injection show that it is cleared from the blood within hours through the reticuloendothelial system (Molenaar et al., 2002), particularly of the liver and spleen, where it is retained for days (Zou et al., 2004), potentially activating marginal-zone B-cell responses. Thus, the filamentous phage is not only a highly immunogenic carrier, but by virtue of activating a range of innate and adaptive immune responses, serves as an excellent model virus-like particle antigen.

## Filamentous Phage as a Therapeutic and Prophylactic Agent in Bacterial Infection, Cancer, and Chronic Disease

Long before the identification of filamentous phage, other types of bacteriophage were already being used for antibacterial therapy in the former Soviet Union and Eastern Europe (reviewed in Sulakvelidze et al., 2001). The filamentous phage, with its non-lytic life cycle, has less obvious clinical uses, despite the fact that the host specificity of *Inovirus* and *Plectrovirus* includes many pathogens of medical importance, including *Salmonella, E. coli, Shigella, Pseudomonas, Clostridium*, and *Mycoplasma* species. In an effort to enhance their bactericidal activity, genetically modified filamentous phage have been used as a “Trojan horse” to introduce various antibacterial agents into cells. M13 and Pf3 phage engineered to express either *BglII* restriction endonuclease (Hagens and Blasi, 2003; Hagens et al., 2004), lambda phage S holin (Hagens and Blasi, 2003) or a lethal catabolite gene activator protein (Moradpour et al., 2009) effectively killed *E. coli* and *Pseudomonas aeruginosa* cells, respectively, with no concomitant release of LPS (Hagens and Blasi, 2003; Hagens et al., 2004). Unfortunately, the rapid emergence of resistant bacteria with modified F pili represents a major and possibly insurmountable obstacle to this approach. However, there are some indications that filamentous phage can exert useful but more subtle effects upon their bacterial hosts that may not result in the development of resistance to infection. Several studies have reported increased antibiotic sensitivity in bacterial populations simultaneously infected with either wild type filamentous phage (Hagens et al., 2006) or phage engineered to repress the cellular SOS response (Lu and Collins, 2009). Filamentous phage f1 infection inhibited early stage, but not mature, biofilm formation in *E. coli* (May et al., 2011). Thus, unmodified filamentous phage may be of future interest as elements of combination therapeutics against certain drug-resistant infections.

**FIGURE 2:**
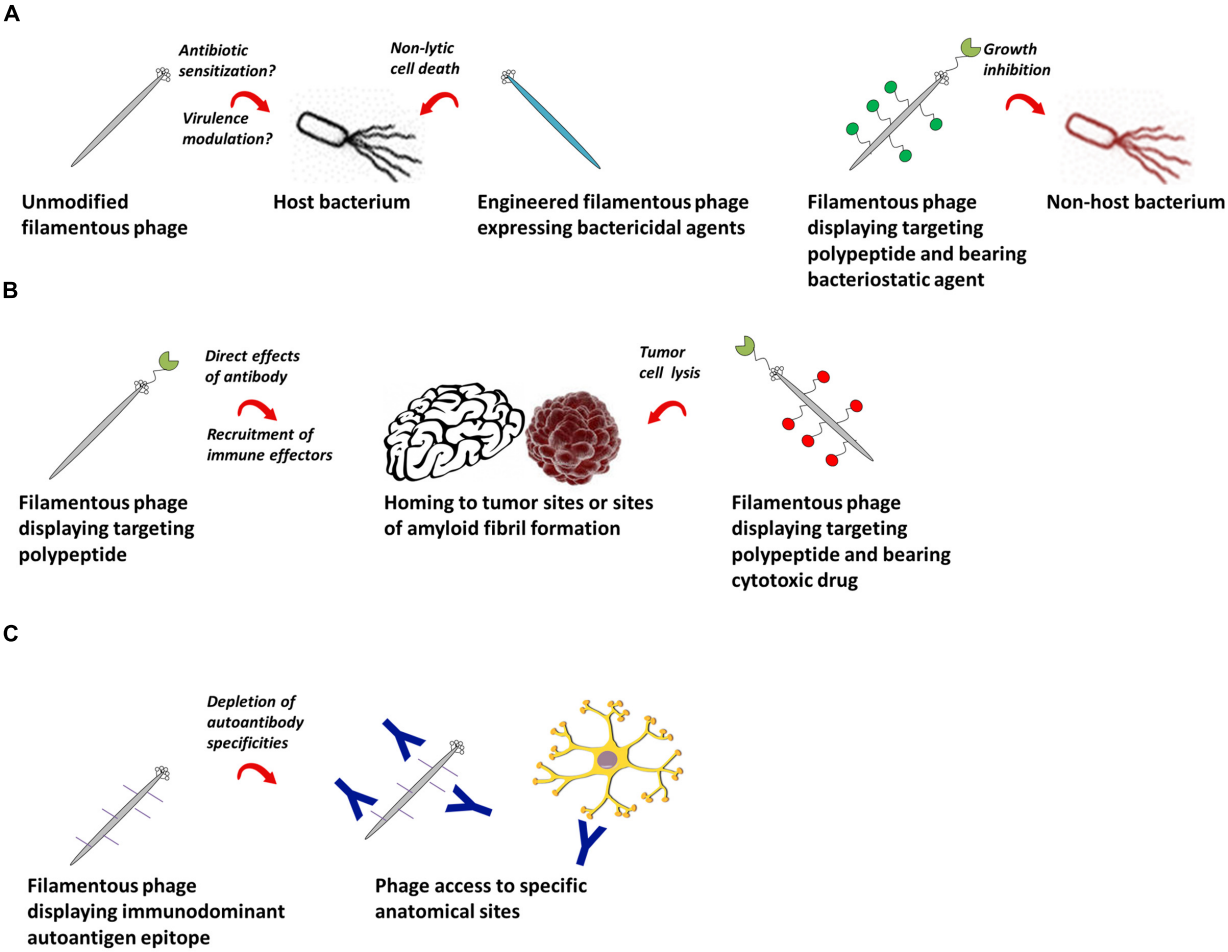
**Potential therapeutic applications of filamentous bacteriophage. (A)** Filamentous phage as a therapeutic against bacterial infections. Left: wild-type or engineered phage bearing genes encoding antibacterial agents can be used as therapeutics against their natural bacterial hosts. Right: filamentous phage displaying antibodies or polypeptides can target bacterial cells outside their natural species tropism. **(B)** Filamentous phage as a therapeutic in cancer and chronic diseases. Phage encoding targeting peptides home specifically to tumor or amyloid fibril sites, where they can have direct effects, recruit immune effector cells, or deliver conjugated cytotoxic drugs. **(C)** Filamentous phage as a therapeutic for autoantibody-mediated autoimmune conditions. Phage displaying immunodominant autoantigen epitopes can selectively deplete autoantibodies of defined specificities.

More advanced therapeutic applications of the filamentous phage emerge when it is modified to express a targeting moiety specific for pathogenic cells and/or proteins for the treatment of infectious diseases, cancer and autoimmunity (**Figure 2**). The first work in this area showed as proof-of-concept that phage encoding a GFP expression cassette and displaying a HER2-specific scFv on all copies of pIII were internalized into breast tumor cells, resulting in GFP expression (Poul and Marks, 1999). M13 or fd phage displaying either a targeting peptide or antibody fragment and tethered to chloramphenicol by a labile crosslinker were more potent inhibitors of *Staphylococcus aureus* growth than high-concentration free chloramphenicol (Yacoby et al., 2006; Vaks and Benhar, 2011). M13 phage loaded with doxorubicin and displaying a targeting peptide on pIII specifically killed prostate cancer cells *in vitro* (Ghosh et al., 2012a). Tumor-specific peptide:pVIII fusion proteins selected from “landscape” phage (Romanov et al., 2001; Abbineni et al., 2010; Fagbohun et al., 2012, 2013; Lang et al., 2014; Wang et al., 2014a) were able to target and deliver siRNA-, paclitaxel-, and doxorubicin-containing liposomes to tumor cells (Jayanna et al., 2010a; Wang et al., 2010a,b,c, 2014b,c; Bedi et al., 2011, 2013, 2014); they were non-toxic and increased tumor remission rates in mouse models (Jayanna et al., 2010b; Wang et al., 2014b,c). Using the B16-OVA tumor model, Eriksson et al. (2007) showed that phage displaying peptides and/or Fabs specific for tumor antigens delayed tumor growth and improved survival, owing in large part to activation of tumor-associated macrophages and recruitment of neutrophils to the tumor site (Eriksson et al., 2009). Phage displaying an scFv against β-amyloid fibrils showed promise as a diagnostic (Frenkel and Solomon, 2002) and therapeutic (Solomon, 2008) reagent for Alzheimer’s disease and Parkinson’s disease due to the unanticipated ability of the phage to penetrate into brain tissue (Ksendzovsky et al., 2012). Similarly, phage displaying an immunodominant peptide epitope derived from myelin oligodendrocyte glycoprotein depleted pathogenic demyelinating antibodies in brain tissue in the murine experimental autoimmune encephalomyelitis model of multiple sclerosis (Rakover et al., 2010). The advantages of the filamentous phage in this context over traditional antibody-drug or protein–peptide conjugates are (i) its ability to carry very high amounts of drug or peptide, and (ii) its ability to access anatomical compartments that cannot generally be reached by systemic administration of a protein.

Unlike most therapeutic biologics, the filamentous phage’s production in bacteria complicates its use in humans in several ways. First and foremost, crude preparations of filamentous phage typically contain very high levels of contaminating LPS, in the range of ~10^2^–10^4^ endotoxin units (EU)/mL (Boratynski et al., 2004; Branston et al., 2015), which have the potential to cause severe adverse reactions. LPS is not completely removed by polyethylene glycol precipitation or cesium chloride density gradient centrifugation (Smith and Gingrich, 2005; Branston et al., 2015), but its levels can be reduced dramatically using additional purification steps such as size exclusion chromatography (Boratynski et al., 2004; Zakharova et al., 2005), polymyxin B chromatography (Grabowska et al., 2000), and treatment with detergents such as Triton X-100 or Triton X-114 (Roehnisch et al., 2014; Branston et al., 2015). These strategies routinely achieve endotoxin levels of <1 EU/mL as measured by the limulus amebocyte lysate (LAL) assay, well below the FDA limit for parenteral administration of 5 EU/kg body weight/dose, although concerns remain regarding the presence of residual virion-associated LPS which may be undetectable. A second and perhaps unavoidable consequence of the filamentous phage’s bacterial production is inherent heterogeneity of particle size and the spectrum of host cell-derived virion-associated and soluble contaminants, which may be cause for safety concerns and restrict its use to high-risk groups.

Many types of bacteriophage and engineered phage variants, including filamentous phage, have been proposed for prophylactic use *ex vivo* in food safety, either in the production pipeline (reviewed in Dalmasso et al., 2014) or for detection of foodborne pathogens post-production (reviewed in Schmelcher and Loessner, 2014). Filamentous phage displaying a tetracysteine tag on pIII were used to detect *E. coli* cells through staining with biarsenical dye (Wu et al., 2011). M13 phage functionalized with metallic silver were highly bactericidal against *E. coli* and *Staphylococcus epidermidis* (Mao et al., 2010). Biosensors based on surface plasmon resonance (Nanduri et al., 2007), piezoelectric transducers (Olsen et al., 2006), linear dichroism (Pacheco-Gomez et al., 2012), and magnetoelastic sensor technology (Lakshmanan et al., 2007; Huang et al., 2009) were devised using filamentous phage displaying scFv or conjugated to whole IgG against *E. coli, Listeria monocytogenes, Salmonella typhimurium*, and *Bacillus anthracis* with limits of detection on the order of 10^2^–10^6^ bacterial cells/mL. Proof of concept has been demonstrated for use of such phage-based biosensors to detect bacterial contamination of live produce (Li et al., 2010b) and eggs (Chai et al., 2012).

## Filamentous Phage as a Scaffold for Bioconjugation and Surface Chemistry

**FIGURE 3:**
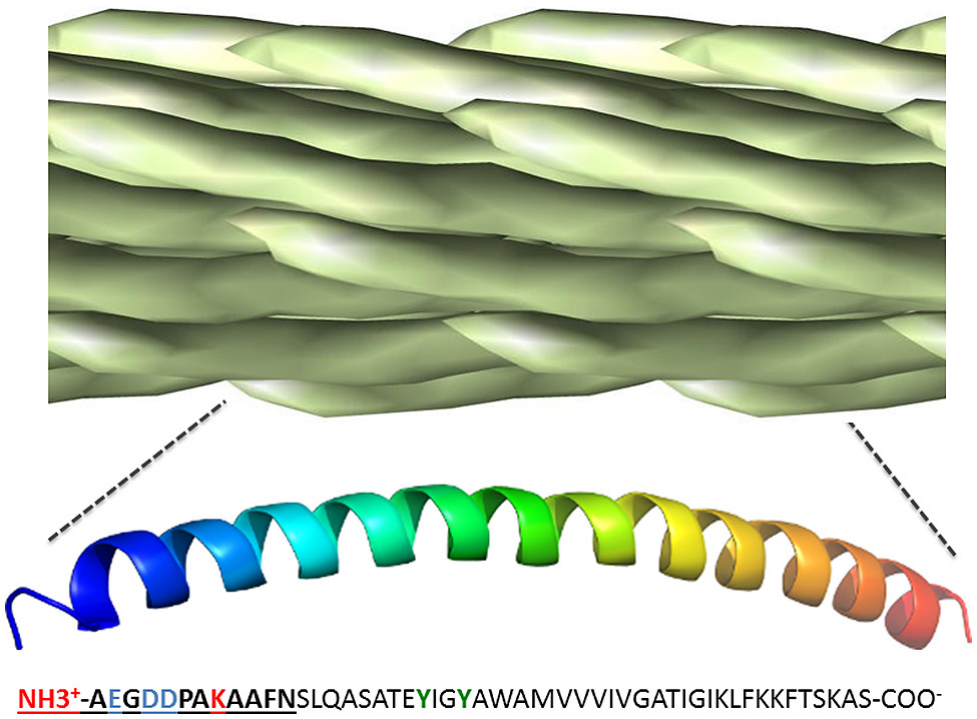
**Chemically addressable groups of the filamentous bacteriophage major coat protein lattice.** The filamentous phage virion is made up of ~2,500–4,000 overlapping copies of the 50-residue major coat protein, pVIII, arranged in a shingle-type lattice. Each monomer has an array of chemically addressable groups available for bioorthogonal conjugation, including two primary amine groups (shown in red), three carboxyl groups (show in blue) and two hydroxyl groups (show in green). The 12 N-terminal residues generally exposed to the immune system for antibody binding are in bold underline. Figure adapted from structural data of Marvin, 1990, freely available in PDB and SCOPe databases.

The filamentous phage particle is enclosed by a rod-like protein capsid, ~1000 nm long and 5 nm wide, made up almost entirely of overlapping pVIII monomers, each of which lies ~27 angstroms from its nearest neighbor and exposes two amine groups as well as at least three carboxyl groups (Henry et al., 2011). The regularity of the phage pVIII lattice and its diversity of chemically addressable groups make it an ideal scaffold for bioconjugation (**Figure 3**). The most commonly used approach is functionalization of amine groups with NHS esters (van Houten et al., 2006, 2010; Yacoby et al., 2006), although this can result in unwanted acylation of pIII and any displayed biomolecules. Carboxyl groups and tyrosine residues can also be functionalized using carbodiimide coupling and diazonium coupling, respectively (Li et al., 2010a). Carrico et al. (2012) developed methods to specifically label pVIII N-termini without modification of exposed lysine residues through a two-step transamination-oxime formation reaction. Specific modification of phage coat proteins is even more easily accomplished using genetically modified phage displaying peptides (Ng et al., 2012) or enzymes (Chen et al., 2007; Hess et al., 2012), but this can be cumbersome and is less general in application.

For more than a decade, interest in the filamentous phage as a building block for nanomaterials has been growing because of its unique physicochemical properties, with emerging applications in magnetics, optics, and electronics. It has long been known that above a certain concentration threshold, phage can form ordered crystalline suspensions (Welsh et al., 1996). Lee et al. (2002) engineered M13 phage to display a ZnS-binding peptide on pIII and showed that, in the presence of ZnS nanoparticles, they self-assemble into highly ordered film biomaterials that can be aligned using magnetic fields. Taking advantage of the ability to display substrate-specific peptides at known locations on the phage filament (Huang et al., 2005; Hess et al., 2012), this pioneering work became the basis for construction of two- and three-dimensional nanomaterials with more advanced architectures, including semiconducting nanowires (Mao et al., 2003, 2004), nanoparticles (Yoo et al., 2006), and nanocomposites (Oh et al., 2012; Chen et al., 2014). Using hybrid M13 phage displaying Co_3_O_4_- and gold-binding peptides on pVIII as a scaffold to assemble nanowires on polyelectrolyte multilayers, Nam et al. (2006) produced a thin, flexible lithium ion battery, which could be stamped onto platinum microband current collectors (Nam et al., 2008). The electrochemical properties of such batteries were further improved through pIII-display of single-walled carbon nanotube-binding peptides (Lee et al., 2009), offering an approach for sustainable production of nanostructured electrodes from poorly conductive starting materials. Phage-based nanomaterials have found applications in cancer imaging (Ghosh et al., 2012b; Yi et al., 2012), photocatalytic water splitting (Nam et al., 2010a; Neltner et al., 2010), light harvesting (Nam et al., 2010b; Chen et al., 2013), photoresponsive technologies (Murugesan et al., 2013), neural electrodes (Kim et al., 2014), and piezoelectric energy generation (Murugesan et al., 2013).

Thus, the unique physicochemical properties of the phage, in combination with modular display of peptides and proteins with known binding specificity, have spawned wholly novel materials with diverse applications. It is worth noting that the unusual biophysical properties of the filamentous phage can also be exploited in the study of structures of other macromolecules. Magnetic alignment of high-concentration filamentous phage in solution can partially order DNA, RNA, proteins, and other biomolecules for measurement of dipolar coupling interactions (Hansen et al., 1998, 2000; Dahlke Ojennus et al., 1999) in NMR spectroscopy.

## Filamentous Phage as an Engine for Experimental Protein Evolution

Because of their large population sizes, short generation times, small genome sizes and ease of manipulation, various filamentous and non-filamentous bacteriophages have been used as models of experimental evolution (reviewed in Husimi, 1989; Wichman and Brown, 2010; Kawecki et al., 2012; Hall et al., 2013). The filamentous phage has additional practical uses in protein engineering and directed protein evolution, due to its unique tolerance of genetic modifications that allow biomolecules to be displayed on the virion surface. First and foremost among these applications is *in vitro* affinity maturation of antibody fragments displayed on pIII. Libraries of variant Fabs and single chain antibodies can be generated *via* random or site-directed mutagenesis and selected on the basis of improved or altered binding, roughly mimicking the somatic evolution strategy of the immune system (Marks et al., 1992; Bradbury et al., 2011). However, other *in vitro* display systems, such as yeast display, have important advantages over the filamentous phage for affinity maturation (although each display technology has complementary strengths; Koide and Koide, 2012), and regardless of the display method, selection of “improved” variants can be slow and cumbersome. Iterative methods have been developed to combine computationally designed mutations (Lippow et al., 2007) and circumvent the screening of combinatorial libraries, but these have had limited success to date.

Recently, Esvelt et al. (2011) developed a novel strategy for directed evolution of filamentous phage-displayed proteins, called phage-assisted continuous evolution (PACE), which allows multiple rounds of evolution per day with little experimental intervention. The authors engineered M13 phage to encode an exogenous protein (the subject for directed evolution), whose functional activity triggers gene III expression from an accessory plasmid; variants of the exogenous protein arise by random mutagenesis during phage replication, the rate of which can be increased by inducible expression of error-prone DNA polymerases. By supplying limiting amounts of receptive *E. coli* cells to the engineered phage variants, Esvelt et al. (2011) elegantly linked phage infectivity and production of offspring with the presence of a desired protein phenotype. Carlson et al. (2014) later showed that PACE selection stringency could be modulated by providing small amounts of pIII independently of protein phenotype, and undesirable protein functions negatively selected by linking them to expression of a truncated pIII variant that impairs infectivity in a dominant negative fashion. PACE is currently limited to protein functions that can be linked in some way to the expression of a gene III reporter, such as protein–protein interaction, recombination, DNA or RNA binding, and enzymatic catalysis (Meyer and Ellington, 2011). This approach represents a promising avenue for both basic research in molecular evolution (Dickinson et al., 2013) and synthetic biology, including antibody engineering.

## Filamentous Phage Ecology

Filamentous bacteriophage have been recovered from diverse environmental sources, including soil (Murugaiyan et al., 2011), coastal fresh water (Xue et al., 2012), alpine lakes (Hofer and Sommaruga, 2001) and deep sea bacteria (Jian et al., 2012), but not, perhaps surprisingly, the human gut (Kim et al., 2011). The environmental “phageome” in soil and water represent the largest source of replicating DNA on the planet, and is estimated to contain upward of 10^30^ viral particles (Ashelford et al., 2003; Chibani-Chennoufi et al., 2004; Suttle, 2005). The few studies attempting to investigate filamentous phage environmental ecology using classical environmental microbiology techniques (typically direct observation by electron microscopy) found that filamentous phage made up anywhere from 0 to 100% of all viral particles (Demuth et al., 1993; Pina et al., 1998; Hofer and Sommaruga, 2001). There was some evidence of seasonal fluctuation of filamentous phage populations in tandem with the relative abundance of free-living heterotrophic bacteria (Hofer and Sommaruga, 2001). Environmental metagenomics efforts are just beginning to unravel the composition of viral ecosystems. The existing data suggest that filamentous phage comprise minor constituents of viral communities in freshwater (Roux et al., 2012) and reclaimed and potable water (Rosario et al., 2009) but have much higher frequencies in wastewater and sewage (Cantalupo et al., 2011; Alhamlan et al., 2013), with the caveat that biases inherent to the methodologies for ascertaining these data (purification of viral particles, sequencing biases) have not been not well validated. There are no data describing the population dynamics of filamentous phage and their host species in the natural environment.

At the individual virus-bacterium level, it is clear that filamentous phage can modulate host phenotype, including the virulence of important human and crop pathogens. This can occur either through direct effects of phage replication on cell growth and physiology, or, more typically, by horizontal transfer of genetic material contained within episomes and/or chromosomally integrated prophage. Temperate filamentous phage may also play a role in genome evolution (reviewed in Canchaya et al., 2003). Perhaps the best-studied example of virulence modulation by filamentous phage is that of *Vibrio cholerae*, whose full virulence requires lysogenic conversion by the cholera toxin-encoding CTXϕ phage (Waldor and Mekalanos, 1996). Integration of CTXϕ phage occurs at specific sites in the genome; these sequences are introduced through the combined action of another filamentous phage, fs2ϕ, and a satellite filamentous phage, TLC-Knϕ1 (Hassan et al., 2010). Thus, filamentous phage species interact and coevolve with each other in addition to their hosts. Infection by filamentous phage has been implicated in the virulence of *Yersinia pestis* (Derbise et al., 2007), *Neisseria meningitidis* (Bille et al., 2005, 2008), *Vibrio parahaemolyticus* (Iida et al., 2001), *E. coli* 018:K1:H7 (Gonzalez et al., 2002), *Xanthomonas campestris* (Kamiunten and Wakimoto, 1982), and *P. aeruginosa* (Webb et al., 2004), although in most of these cases, the specific mechanisms modulating virulence are unclear. Phage infection can both enhance or repress virulence depending on the characteristics of the phage, the host bacterium, and the environmental milieu, as is the case for the bacterial wilt pathogen *Ralstonia solanacearum* (Yamada, 2013). Since infection results in downregulation of the pili used for viral entry, filamentous phage treatment has been proposed as a hypothetical means of inhibiting bacterial conjugation and horizontal gene transfer, so as to prevent the spread of antibiotic resistance genes (Lin et al., 2011).

Finally, the filamentous phage may also play a future role in the preservation of biodiversity of other organisms in at-risk ecosystems. Engineered phage have been proposed for use in bioremediation, either displaying antibody fragments of desired specificity for filtration of toxins and environmental contaminants (Petrenko and Makowski, 1993), or as biodegradable polymers displaying peptides selected for their ability to aggregate pollutants, such as oil sands tailings (Curtis et al., 2011, 2013). Engineered phage displaying peptides that specifically bind inorganic materials have also been proposed for use in more advanced and less intrusive mineral separation technologies (Curtis et al., 2009).

## Conclusion and Future Perspectives

The filamentous phage represents a highly versatile organism whose uses extend far beyond traditional phage display and affinity selection of antibodies and polypeptides of desired specificity. Its high immunogenicity and ability to display a variety of surface antigens make the phage an excellent particulate vaccine carrier, although its bacterial production and preparation heterogeneity likely limits its applications in human vaccines at present, despite being apparently safe and well-tolerated in animals and people. Unanticipated characteristics of the phage particle, such as crossing of the blood–brain barrier and formation of highly ordered liquid crystalline phases, have opened up entirely new avenues of research in therapeutics for chronic disease and the design of nanomaterials. Our comparatively detailed understanding of the interactions of model filamentous phage with their bacterial hosts has allowed researchers to harness the phage life cycle to direct protein evolution in the lab. Hopefully, deeper knowledge of phage–host interactions at an ecological level may produce novel strategies to control bacterial pathogenesis. While novel applications of the filamentous phage continue to be developed, the phage is likely to retain its position as a workhorse for therapeutic antibody discovery for many years to come, even with the advent of competing technologies.

## Author Contributions

KH and JS conceived and wrote the manuscript. MA-G read the manuscript and commented on the text.

## Conflict of Interest Statement

The authors declare that the research was conducted in the absence of any commercial or financial relationships that could be construed as a potential conflict of interest.
